# To the Delegates to the American Medical Association

**Published:** 1886-04

**Authors:** Le Grand Atwood

**Affiliations:** Chairman Committee of Arrangements


To The Delegates to the American Medical Association.

The rates given to the Delegates to the American Medical Association Meeting, May 4th, in St. Louis, have been fixed by the different railroad committees of the country at one and one-third fares for the round trip. Delegates must pay full fare coming, and will receive, on application, from the agent at starting point, a certificate, which, when signed by the Chairman of the Local Committee of Arrangements, will entitle them to the reduced return rate.

No reduced return ticket will be issued, unless the purchaser can show a certificate issued by the agent from whom he purchased the going ticket, and signed by the Chairman of the Committee of Arrangements. Le Grand Atwood,

*Chairman Committee of Arrangements.*

---

We call especial attention to the article on Urethane by Henri Huchard, translated by Dr. Campbell. Urethane certainly promises to be the hypnotic of the future, if half that has been said of it is true. It seems to stand the tests of repeated experiments, and the long-felt want of a hypnotic for the very weak and very young seems to be supplied. Its many advantages over all others are set forth in the article, which deserves careful study.

